# Some Failures Attending the Use of Weld's Chemico-Metallic Method of Filling Root Canals

**Published:** 1898-05

**Authors:** H. B. Hinman


ARTICLE VI.

### SOME FAILURES ATTENDING THE USE OF WELD'S CHEMICO-METALLIC METHOD OF FILLING ROOT CANALS.

DR. H. B. HINMAN.

On the 10th of last July I removed the pulp from a second upper left bicuspid for Miss G., age twenty-two, and filled the root-canal with a Weld's chemico metallic point, following the directions given by the inventor.

The tooth was then filled with amalgam. On August 16th the patient returned with a severe alveola abscess upon the tooth. The filling and point were removed, an incision made through the gum and alveola process, and the abscess treated and cured in the usual way, the root being filled this time with chloro-percha and gutta percha cones.

On July 15th an upper right first bicuspid was treated in a similar manner for the same patient—the pulp being thoroughly removed from both roots, and the roots being filled with the points. A gold crown was then inserted. The tooth gave no trouble until the middle of December, when it began to get sore. When the crown was removed on the 29th inst., there was a copious flow of rather thick pus from the lingual root-canal as soon as the point was withdrawn. At the same time at which I was doing the work on the teeth above mentioned, I also devitalized the upper left first bicuspid and lower right second bicuspid and filled the root-canal with chloro-percha and gutta-percha cones, and they have given no trouble whatever. I used about thirty of the points during July and August, and have had one other similar experience.

While the others have given no trouble as yet, I am afraid that it will be. *Au revoir*, but not "good-bye."—*Ohio Denial Journal.*

